# Comparison of the safety and efficacy of remimazolam for sedation during bronchoscopy: a meta-analysis of randomized controlled trials

**DOI:** 10.7717/peerj.20552

**Published:** 2026-01-09

**Authors:** Yupei Yuan, Chunlei Chang, Jing Zhang, Liang Liang

**Affiliations:** 1 Department of Cardiology, Renmin Hospital of Wuhan University, Wuhan, China; 2 The Fourth Clinical Medical College of Xinjiang Medical University, Urumqi, China; 3 Department of Psychology, The Fourth Affiliated Hospital of Xinjiang Medical University, Xinjiang Medical University, Urumqi, China

**Keywords:** Remimazolam, Bronchoscopy, Meta-analysis

## Abstract

**Objective:**

To evaluate the comparative efficacy and safety of remimazolam *vs*. established sedatives (dexmedetomidine, propofol, midazolam) for sedation during bronchoscopy.

**Methods:**

A systematic review and meta-analysis of randomized controlled trials (RCTs) was conducted according to PRISMA guidelines and Cochrane Handbook recommendations (PROSPERO CRD420251071986). Databases (EMBASE, PubMed, Scopus, Cochrane Central, Web of Science) were searched from inception to May 14, 2025. Included studies were RCTs comparing intravenous remimazolam to comparator sedatives in adults (≥18 years) undergoing bronchoscopy. Primary outcome was procedural success rate (completion without rescue sedation). Secondary outcomes included onset time, wake-up time, procedure duration, patient satisfaction, and adverse events (hypotension, hypoxemia, tachycardia, bradycardia, hypertension). Risk of bias was assessed using RoB 2. Data were pooled using random-effects models, reporting mean differences (MD) or odds ratios (OR) with 95% confidence intervals (CI).

**Results:**

13 RCTs (*n* = 2,002 patients) were included. Remimazolam demonstrated: Procedural success: Significantly higher success rates *vs*. dexmedetomidine (OR 2.87, 95% CI [1.13–7.29], *P* = 0.03; I^2^ = 62%) and *vs*. midazolam (OR 3.65, 95% CI [1.40–9.49], *P* = 0.008; absolute rates 77.1% *vs*. 3.6%). Comparable success rates to propofol (OR 0.55, 95% CI [0.11–2.61], *P* = 0.45; rates ~98% each). Onset/Recovery: Faster onset *vs*. dexmedetomidine (MD −2.01 min, 95% CI [−2.08 to −1.93], *P* < 0.001; I^2^ = 0%). Shorter wake-up time *vs*. dexmedetomidine (MD −1.84 min, 95% CI [−3.31 to −0.37], *P* = 0.01) and *vs*. midazolam (MD −4.72 min, 95% CI [−8.05 to −1.39], *P* = 0.005). Comparisons with propofol were inconclusive due to heterogeneity. Safety: Significantly lower risk of hypotension *vs*. propofol (OR 0.35, 95% CI [0.23–0.55], *P* < 0.00001) and hypoxemia *vs*. dexmedetomidine (OR 0.41, 95% CI [0.18–0.96], *P* = 0.04). Significantly higher risk of tachycardia *vs*. dexmedetomidine (OR 3.01, 95% CI [1.37–6.60], *P* = 0.006). No significant safety differences *vs*. midazolam. Patient satisfaction was equivalent to propofol.

**Conclusion:**

Remimazolam is a highly effective and safe sedative for bronchoscopy. It offers superior procedural success and faster recovery compared to dexmedetomidine and midazolam, while matching the high success rate of propofol. Its key safety advantage is significantly reduced hypotension risk compared to propofol, making it particularly suitable for vulnerable patients. Remimazolam represents a valuable addition to sedation options for this procedure.

## Introduction

Bronchoscopy, both diagnostic and therapeutic, is a cornerstone procedure in respiratory medicine. While essential for managing conditions such as lung cancer, infections, interstitial lung diseases, and airway obstruction, it is inherently invasive and frequently associated with significant patient discomfort, anxiety, pain, coughing, and gagging (Papavasileiou et al., 2025; Peralta & Shadid, 2025). Adequate sedation and analgesia are therefore crucial not only for patient tolerance and procedural satisfaction but also for optimizing operator conditions, facilitating successful examination or intervention, and minimizing procedure-related complications (Kim et al., 2025; Paez & Maldonado, 2025).

Traditionally, benzodiazepines like midazolam, often combined with opioids, and the hypnotic agent propofol have been the mainstays for sedation during bronchoscopy (Moraru et al., 2025). Midazolam offers anxiolysis and amnesia but has drawbacks including variable pharmacokinetics, prolonged recovery times, and the risk of respiratory depression, especially in vulnerable populations or when combined with opioids (Bauer et al., 2004). Propofol provides rapid onset and offset, facilitating quicker recovery, but carries a significant risk of dose-dependent hypotension and respiratory depression, often necessitating administration by anesthesia personnel and limiting its use in certain settings or by non-anesthesiologists (Kotani et al., 2008; Marik, 2004). This safety profile is particularly relevant in patients with significant comorbidities, which are common in the bronchoscopy population. The quest for an ideal sedative agent—characterized by rapid onset, predictable pharmacokinetics, minimal cardiorespiratory effects, a favorable safety profile, and the potential for flexible administration—remains a significant clinical need.

Remimazolam (CNS 7056), a novel, ultra-short-acting benzodiazepine, has emerged as a promising candidate to address these limitations. It acts as a positive allosteric modulator of the GABA_A receptor (Yang et al., 2024). Its key differentiating features are its rapid metabolism by tissue esterases to an inactive metabolite (CNS 7054), leading to a predictable and short duration of action independent of hepatic cytochrome P450 enzymes, and its availability of a specific reversal agent (flumazenil) (Abramovich, Lee & Bilotta, 2025; Stojanovic & Jankovic, 2025). These properties theoretically offer advantages: rapid titration to desired effect, minimal accumulation, quick recovery even after prolonged infusion, and a potentially improved hemodynamic and respiratory stability profile compared to midazolam and propofol (Pingel et al., 2025). Initial studies and its established use in procedural sedation and general anesthesia suggest its efficacy and safety.

Consequently, remimazolam has garnered increasing interest for sedation during bronchoscopy. Several randomized controlled trials (RCTs) and observational studies have investigated its use in this specific context, comparing it primarily to midazolam or propofol (Chai et al., 2025; Kim et al., 2023). While individual studies often report favorable outcomes for remimazolam regarding efficacy endpoints (*e.g*., sedation success rate, time to onset, recovery times) and safety (*e.g*., incidence of hypotension, hypoxia, injection pain), the evidence remains scattered. Variations in study design, patient populations, dosing regimens, comparator agents, and definitions of outcomes make it challenging to draw definitive conclusions about its overall comparative profile based on single studies. Furthermore, the power of individual trials to detect differences in less common but clinically significant adverse events may be limited.

Therefore, a comprehensive synthesis of the existing evidence is imperative. This meta-analysis aims to rigorously evaluate and quantitatively synthesize the current evidence on the efficacy and safety of remimazolam specifically for sedation during bronchoscopy. We will assess critical outcomes including procedural success rates, sedation quality, times to onset and recovery, hemodynamic stability (incidence of hypotension), respiratory safety (incidence of hypoxia/bradypnea), and other adverse events, comparing remimazolam primarily to midazolam and propofol. By pooling data from available RCTs, this study seeks to provide clinicians, anesthesiologists, and bronchoscopists with a higher level of evidence to inform sedation choices and optimize patient care during this essential procedure.

## Materials and Methods

This meta-analysis was prospectively registered with the International Prospective Register of Systematic Reviews (PROSPERO) (Registration No: CRD 420251071986). The conduct and reporting strictly adhered to the Preferred Reporting Items for Systematic Reviews and Meta-Analyses (PRISMA) 2020 statement and the Cochrane Handbook for Systematic Reviews of Interventions.

### Search strategy

A systematic and comprehensive literature search was performed independently by two investigators (Liang and Yuan) from inception until 14 May 2025 in the following databases: EMBASE, PubMed, Scopus, Cochrane Central, and Web of Science databases. In cases of disagreement, researchers will resolve the issue *via* discussion. Failing resolution, the third author (Chang) shall serve as the final arbiter. Search strategies utilized a combination of controlled vocabulary (MeSH terms in PubMed, Emtree in EMBASE) and free-text keywords related to: “remimazolam” OR “CNS 7056” AND “bronchoscop” OR “bronchial endoscop” OR “airway endoscop” AND “randomized controlled trial” OR “RCT” OR “random” OR “controlled trial”. No language restrictions were applied initially. Filters for RCTs were used where available.

### Eligibility criteria and study selection

We included all studies that met following Population, Intervention, Comparator, Outcomes, Study Design (PICOS). (1) Population (P): Adult patients (≥18 years) undergoing diagnostic or therapeutic bronchoscopy (flexible or rigid) under sedation. Sedation using intravenous remimazolam besylate (any dose or regimen); (2) Intervention (I): Sedation using intravenous remimazolam besylate (any dose or regimen); (3) Comparator (C): Midazolam (any dose/regimen), Propofol (any dose/regimen, including TCI or bolus), Placebo (if applicable), Other active comparators (*e.g*., dexmedetomidine—analyzed separately if sufficient data); (4) Outcomes (O): RCTs reporting at least one relevant outcome. (5) Study Design (S): Randomized Controlled Trials (RCTs) published as full-text articles in peer-reviewed journals. Conference abstracts, non-randomized studies, case reports, reviews, and animal studies were excluded.

All identified records from the database searches were imported into EndNote X9 (Clarivate Analytics) and duplicates removed. During the initial screening phase, titles and abstracts were assessed for relevance by two independent reviewers. Potentially eligible studies then underwent a full-text review, where the same reviewers applied the pre-specified inclusion and exclusion criteria (PICOS) independently to make the final selection. Disagreements at any stage were resolved through discussion or consultation with a third reviewer. The selection process was documented using a PRISMA flow diagram.

### Outcome measures

The primary outcome was procedural success rate (completion of the intended bronchoscopy procedure without requiring rescue sedative or alternative sedation). Secondary outcomes were anesthesia onset time, procedure duration, wake up time, patient satisfaction and adverse events (bradycardia, hypertension, hypotension, tachycardia and hypoxemia).

### Data extraction

Data extraction was performed independently by two reviewers (initials) using a pre-piloted, standardized electronic form. Discrepancies were resolved by consensus or third-reviewer arbitration. Extracted data included: study characteristics, participant characteristics, intervention details, comparator details and outcome data.

### Risk of bias assessment

The risk of bias in individual RCTs was assessed independently by two reviewers using the revised Cochrane Risk of Bias tool for randomized trials (RoB 2). The Cochrane risk of bias tool detects the following types of bias: (1) Bias arising from the randomization process; (2) Bias due to inadequate allocation concealment; (3) Bias related to blinding of participants and personnel; (4) Bias in outcome assessment; (5) Bias from incomplete outcome data; (6) Bias in selective reporting of results. Two independent reviewers judged each domain as ‘Low risk’, ‘unclear’, or ‘High risk’ based on pre-defined criteria.

### Statistical analysis

We performed all statistical syntheses using Review Manager (RevMan) v5.4 and R software. We employed a random-effects model as our primary approach to account for anticipated heterogeneity. We analyzed continuous data by calculating mean differences (MD) and dichotomous data by estimating risk ratios (RR), both reported with 95% confidence intervals. We assessed statistical heterogeneity using the I^2^ statistic, which we interpreted as low (0–40%), moderate (30–60%), substantial (50–90%), or considerable (75–100%). We conducted sensitivity analyses using a fixed-effect model for outcomes with low heterogeneity (I^2^ < 40%). We defined a two-tailed *P*-value of less than 0.05 as statistically significant.

## Results

**Figure 1:**
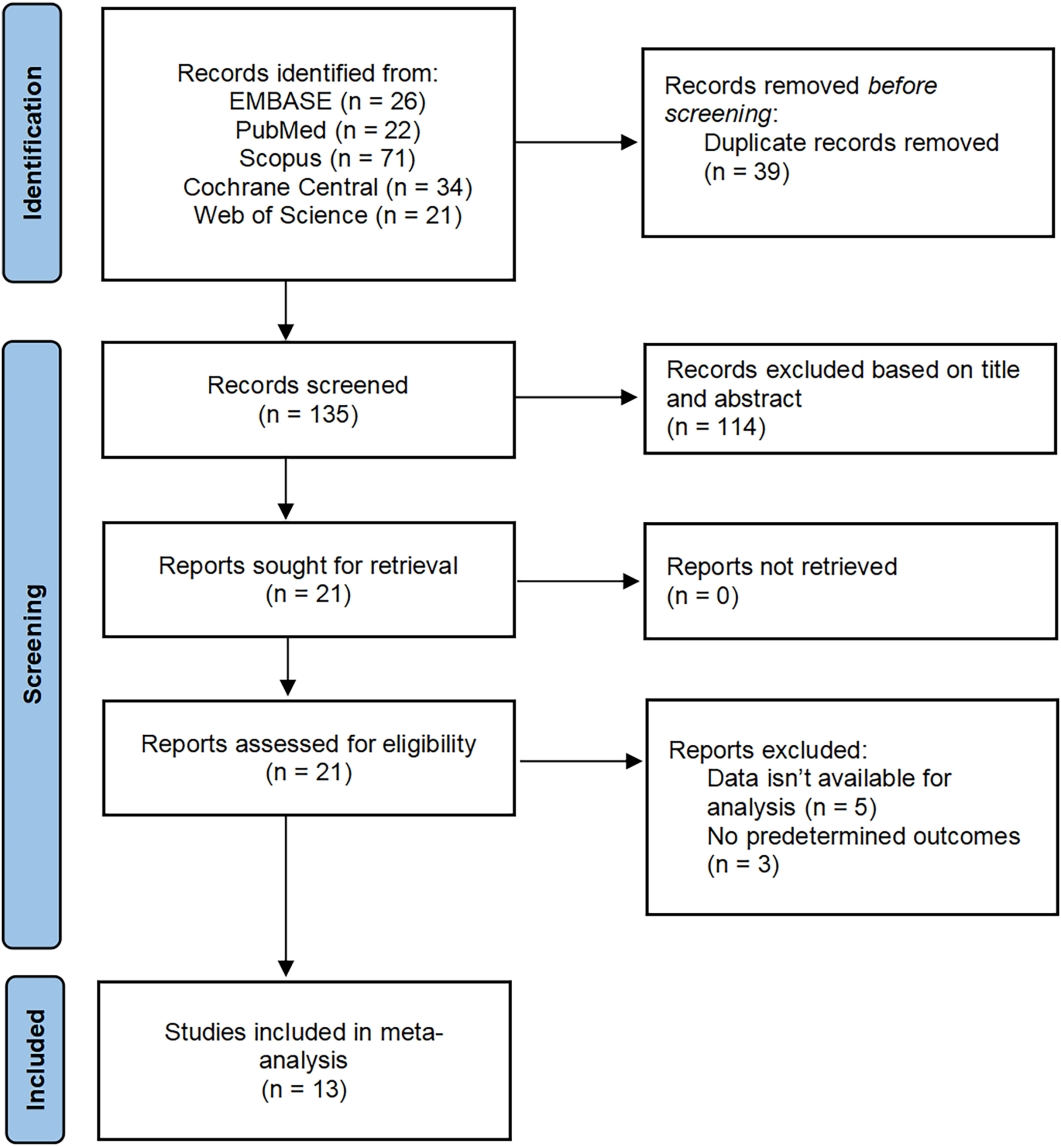
PRISMA flow diagram of study selection.

A total of 174 studies were identified through searches of EMBASE, PubMed, Scopus, Cochrane Central, and Web of Science databases. After eliminating 39 duplicate articles, 114 were removed by reading the title and abstract, and 8 were removed after reading the full text. Finally, 13 met the eligibility criteria and were included in the meta-analysis Fig. 1.

### Characteristics of trials

This meta-analysis incorporated thirteen randomized controlled trials (RCTs) (Chai et al., 2025; Xu et al., 2024; Zhang et al., 2023; Zhou et al., 2024; Pastis et al., 2019; Luo et al., 2025; Chen et al., 2024; Wu et al., 2024; Gao et al., 2022; Kim et al., 2023; Chen et al., 2022; Pan et al., 2023; Zhou et al., 2022), enrolling a total of 2,002 participants, published between 2019 and 2024. All included studies were peer-reviewed publications in English. Key methodological and clinical characteristics of the trials are comprehensively summarized in Table 1.

**Table 1:** Characteristics of included randomized controlled trials comparing remimazolam with other sedatives for bronchoscopy sedation.

Clinical trials	Age (Years)	Sex (M/F)	ASA (I/II/III)	BMI (kg/m^2^)	Number of patients	Dose of opioids (μg/kg)
**Zhou 2024**						
Remimazolam	60 (52–66)	126/56	6/162/14	22.60 ± 2.74	182	100–200
Dexmedetomidine	61 (55–65)	116/66	10/164/8	22.24 ± 2.78	182	0.4–0.8
**Zhang 2023**						
Remimazolam	63.57 ± 13.32	48/48	–	21.26 ± 3.81	96	200
Propofol	65.11 ± 13.46	44/52	–	21.81 ± 3.70	96	1,500
**Xu 2024**						
Remimazolam	60.7 ± 4.3	40/20	22/38/0	24.1 ± 1.9	60	1,000–2,000
Dexmedetomidine	60.9 ± 4.2	41/19	23/37/0	24.5 ± 2.3	60	0.5
**Luo 2025**						
Remimazolam	71.3 ± 4.0	24/9	0/29/4	24.8 ± 5.7	33	200
Propofol	71.5 ± 3.7	21/12	0/27/6	25.2 ± 6.1	33	1,500
**Chai 2025**						
Remimazolam	70.2 ± 4.1	17/13	2/28/0	22.4 ± 2.7	30	200
Propofol	69.7 ± 4.4	18/12	1/29/0	23.4 ± 2.7	30	2,000
**Wu 2024**						
Remimazolam	70.37 ± 4.07	26/20	6/33/7	22.09 ± 3.65	46	135
Midazolam	69.21 ± 3.59	29/19	7/32/9	22.19 ± 3.06	48	45
**Chen 2024**						
Remimazolam	43.33 ± 15.33	23/7	9/20/1	20.70 ± 2.77	30	93
Dexmedetomidine	48.97 ± 10.01	24/6	2/25/2	21.63 ± 3.94	30	0.6
**Chen 2022**						
Remimazolam	57.05 ± 5.60	55/18	25/48/0	24.58 ± 2.19	73	1,000–2,000
Dexmedetomidine	56.05 ± 5.99	53/20	27/46/0	25.11 ± 2.02	73	0.2–0.7
**Zhou 2022**						
Remimazolam	49.7 ± 13.4	72/83	27/126/2	23.0 ± 2.62	155	200
Propofol	51.9 ± 11.8	82/73	19/135/1	23.5 ± 2.75	155	2,000
**Pan 2022**						
Remimazolam	61.13 ± 8.62	12/3	0/7/7	20.08 ± 3.81	15	400
Propofol	61.13 ± 7.24	15/0	0/11/4	21.73 ± 2.89	15	1,500
**Pastis 2019**						
Remimazolam	62.7 ± 12.09	139/164	–	28.4 ± 6.39	303	5,000
Midazolam	61.5 ± 14.03	35/34	–	28.0 ± 5.79	69	1,000–1,750
**Gao 2022**						
Remimazolam	59.9 ± 7.3	17/13	1/29/0	23.5 ± 2.8	30	6,000
Propofol	58.9 ± 10.8	22/8	2/28/0	23.7 ± 3.9	30	2,000
**Kim 2023**						
Remimazolam	65 (55–74)	31/18	–	23.9 (21.0–25.5)	49	2,500
Midazolam	68 (60–75)	30/21	–	21.9 (19.8–24.0)	51	500

**Note:**

Abbreviations: RCT, randomized controlled trial; ASA, American Society of Anesthesiologists; BMI, body mass index; NR, not reported; SD, standard deviation; IQR, interquartile range (Zhou et al., 2024; Zhang et al., 2023; Xu et al., 2024; Chai et al., 2025; Wu et al., 2024; Chen et al., 2024, 2022; Zhou et al., 2022; Pan et al., 2023; Pastis et al., 2019; Gao et al., 2022; Kim et al., 2023; Luo et al., 2025.)

### Risk of bias in included studies

**Figure 2:**
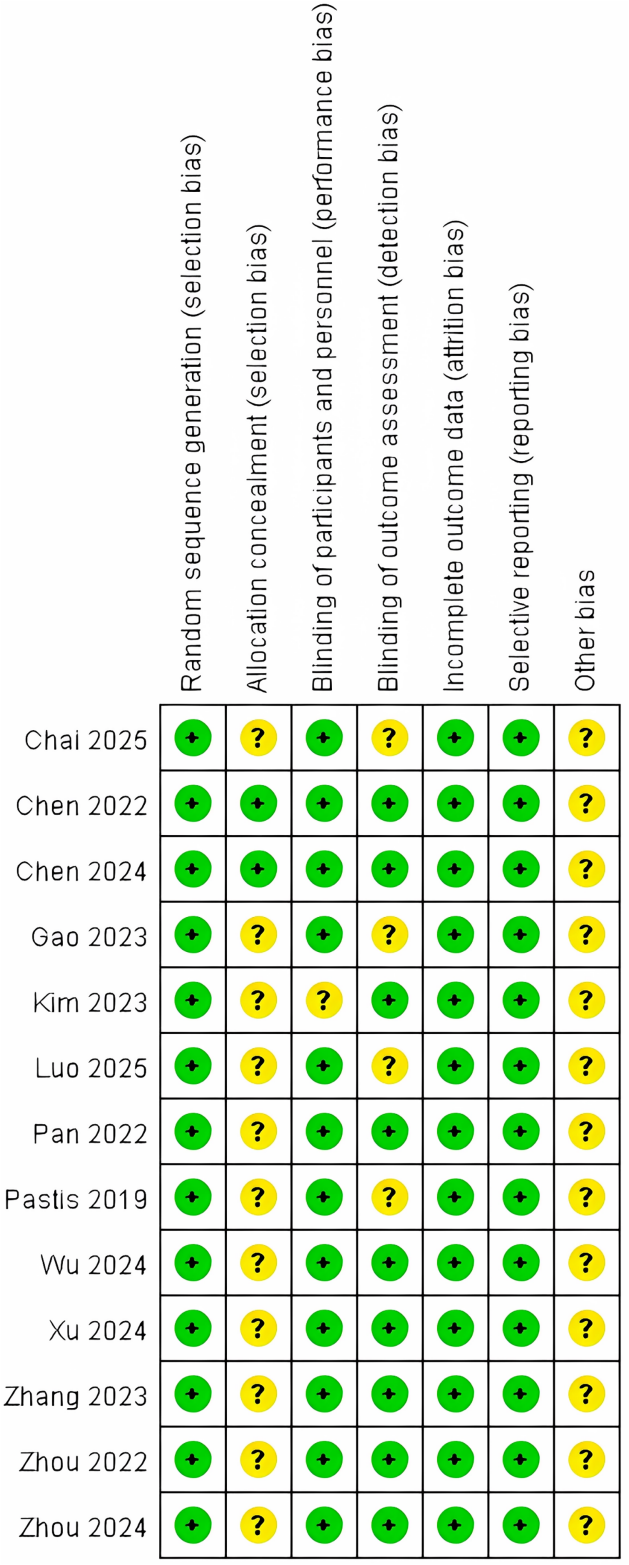
Risk of bias assessment for included studies using the Cochrane RoB 2 tool (Zhou et al., 2024; Zhang et al., 2023; Xu et al., 2024; Chai et al., 2025; Wu et al., 2024; Chen et al., 2024, 2022; Zhou et al., 2022; Pan et al., 2023; Pastis et al., 2019; Gao et al., 2022; Kim et al., 2023; Luo et al., 2025).

Methodological quality was appraised for all thirteen included studies using the Cochrane Risk of Bias 2.0 (RoB 2) tool implemented in Review Manager 5.4. Independent dual-reviewer assessment demonstrated a predominantly low overall risk of bias across studies, with no domains judged as high risk. Domain-specific judgments are detailed in Fig. 2.

### Effect of interventions

#### Primary outcomes

In this meta-analysis evaluating procedural success rates of remimazolam *vs*. comparator sedatives:

*vs*. dexmedetomidine (four RCTs):

Remimazolam demonstrated significantly higher success rates (pooled OR: 2.87; 95% CI [1.13–7.29]; *P* = 0.03), with 2.87-fold greater odds of success. Moderate heterogeneity was observed (I^2^ = 62%, *P* = 0.05), attributable to clinical variance across studies. All trials consistently favored remimazolam, with Zhou et al. (2024) showing the largest effect (OR: 7.12; 95% CI [3.98–13.06]).

*vs*. propofol (six RCTs identified; three analyzable):

No significant difference was found (pooled OR: 0.55; 95% CI [0.11–2.61]; *P* = 0.45). Success rates were high in both groups (remimazolam: 98.2% [214/218]; propofol: 99.1% [216/218]). Three studies (Gao et al., 2022; Pan et al., 2023; Zhang et al., 2023) were excluded due to unreported data. No heterogeneity existed (I^2^ = 0%, *P* = 0.86).

*vs*. midazolam (three RCTs):

Remimazolam significantly increased success rates (pooled OR: 3.65; 95% CI [1.40–9.49]; *P* = 0.008), with absolute rates of 77.1% (307/398) *vs*. 3.6% (6/168). Substantial heterogeneity (I^2^ = 80%, Tau^2^ = 0.57, *P* = 0.006) was driven by varying patient populations. Sensitivity analysis excluding (Pastis et al., 2019) eliminated heterogeneity (I^2^ = 0%) while maintaining significance (OR: 2.22; 95% CI [1.25–3.94]; *P* = 0.006), confirming robust superiority.

**Figure 3:**
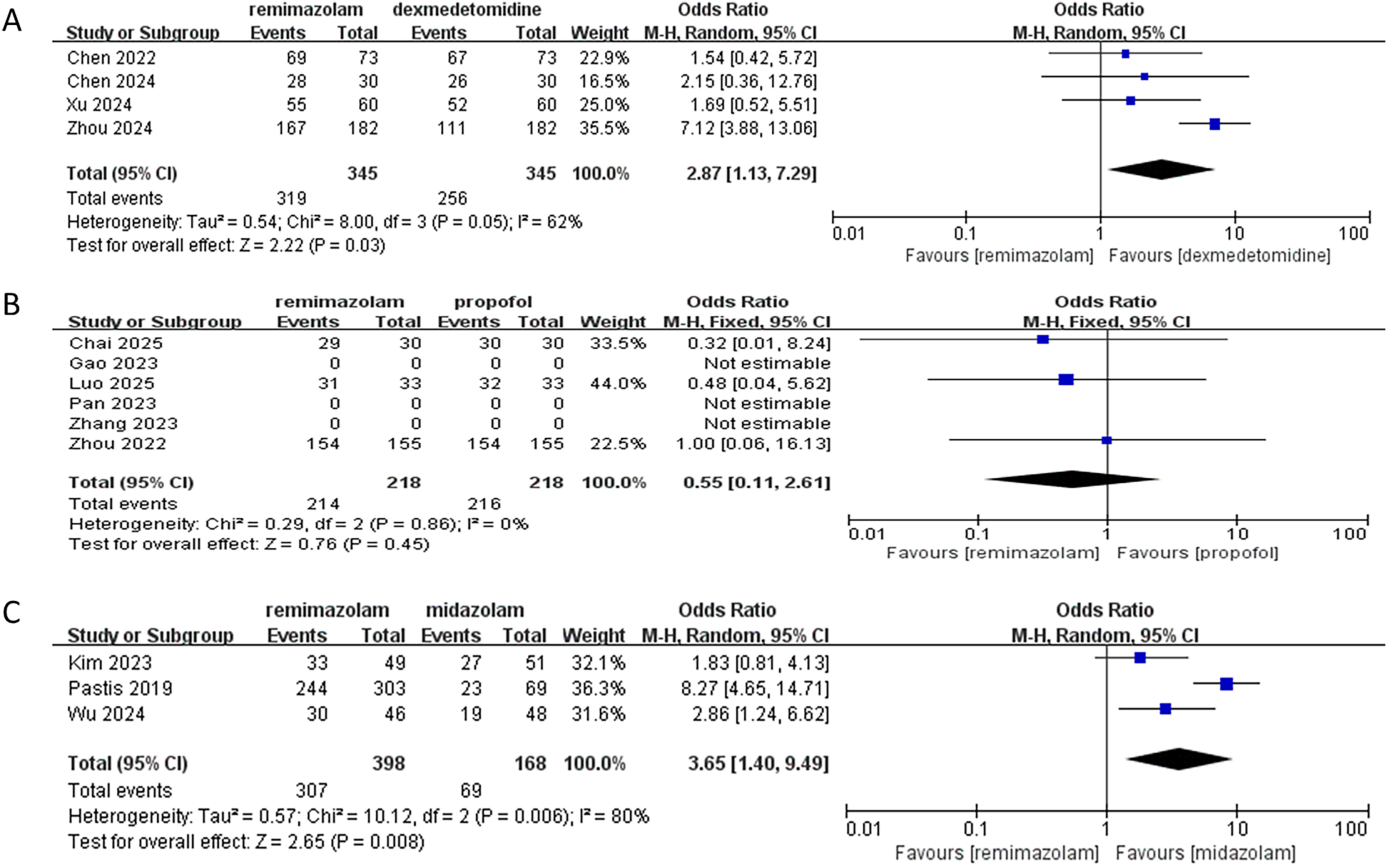
Forest plot comparing procedural success rates of remimazolam *vs*. (A) dexmedetomidine, (B) propofol, and (C) midazolam (Zhou et al., 2024; Zhang et al., 2023; Xu et al., 2024; Chai et al., 2025; Wu et al., 2024; Chen et al., 2024, 2022; Zhou et al., 2022; Pan et al., 2023; Pastis et al., 2019; Gao et al., 2022; Kim et al., 2023; Luo et al., 2025).

The synthesis demonstrates that remimazolam significantly improves procedural success *vs*. dexmedetomidine and midazolam, but exhibits comparable efficacy to propofol (Fig. 3). The high success rates with propofol (>98%) suggest it remains a highly effective alternative, while remimazolam’s superiority over midazolam is clinically substantial (>70% absolute difference). Heterogeneity necessitates cautious interpretation for dexmedetomidine and midazolam comparisons.

### Secondary outcomes

This meta-analysis quantitatively compares the novel benzodiazepine analog remimazolam against three established agents—dexmedetomidine, propofol and midazolam—across four critical endpoints: anesthesia onset time, wake-up time, procedure duration, and patient satisfaction.

**Figure 4:**
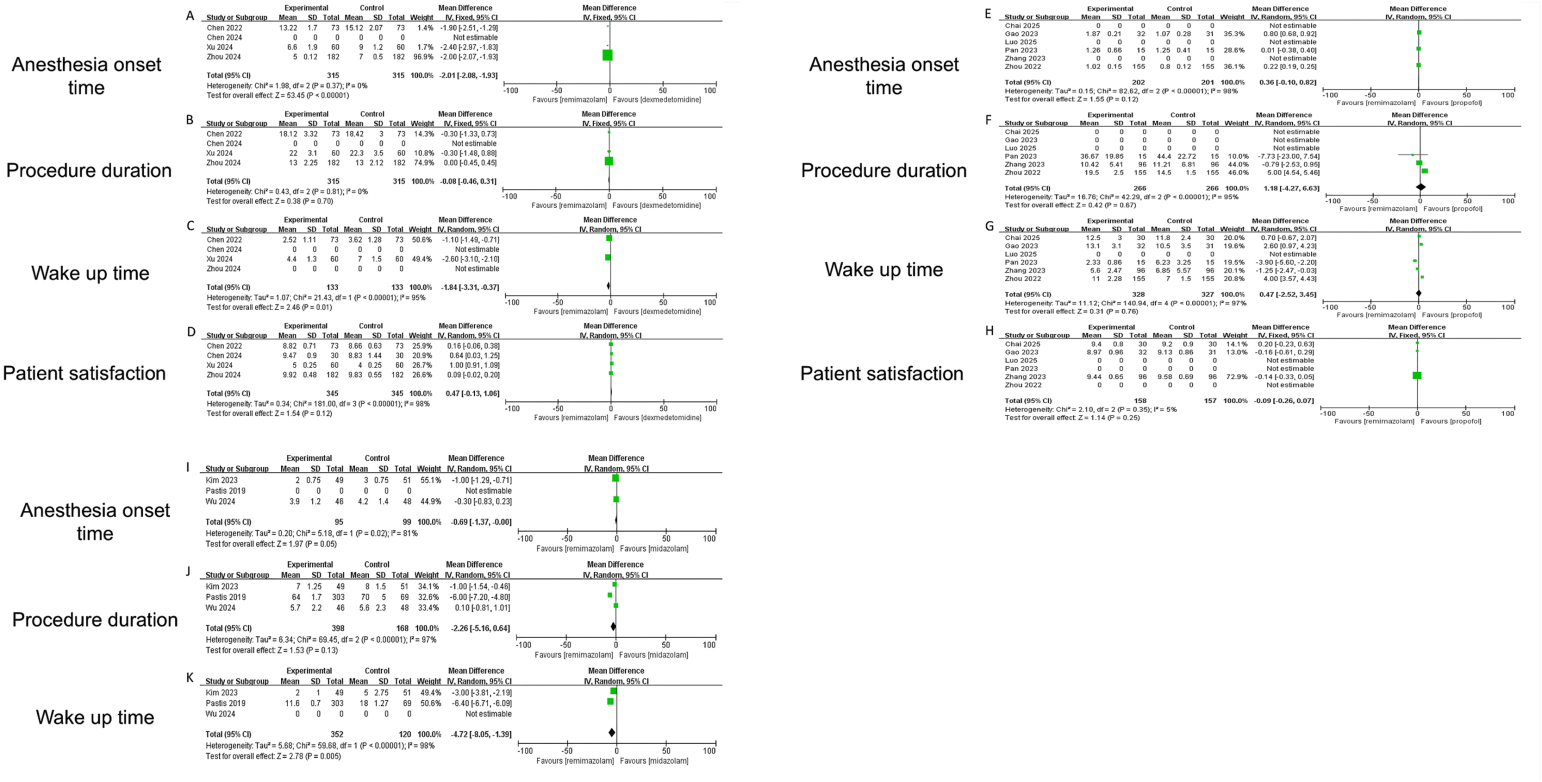
Forest plots comparing secondary outcomes of remimazolam *vs*. dexmedetomidine, propofol, and midazolam: Anesthesia onset time, Wake-up time, Procedure duration, and Patient satisfaction (Zhou et al., 2024; Zhang et al., 2023; Xu et al., 2024; Chai et al., 2025; Wu et al., 2024; Chen et al., 2024; Chen et al., 2022; Zhou et al., 2022; Pan et al., 2023; Pastis et al., 2019; Gao et al., 2022; Kim et al., 2023; Luo et al., 2025).

Remimazolam demonstrated significant advantages in key procedural metrics compared to established sedatives. For anesthesia onset time, remimazolam was markedly faster than dexmedetomidine (mean difference (MD) −2.01 min, 95% CI [−2.08 to −1.93]; *P* < 0.001; I^2^ = 0%) (Fig. 4A), though no significant differences were observed *vs*. propofol (MD 0.36 min, *P* = 0.12) (Fig. 4E) or midazolam (MD −0.69 min, *P* = 0.06) (Fig. 4I), with high heterogeneity noted for the latter comparisons (I^2^ = 81–98%). Recovery (wake-up) time was significantly shorter with remimazolam than both dexmedetomidine (MD −1.84 min, 95% CI [−3.31 to −0.37]; *P* = 0.01) (Fig. 4C) and midazolam (MD −4.72 min, 95% CI [−8.05 to −1.39]; *P* = 0.005) (Fig. 4K), despite substantial heterogeneity (I^2^ ≥ 95%), while comparison with propofol was inconclusive due to extreme heterogeneity (I^2^ = 97%) (Fig. 4G) and opposing inter-study effects. Procedure duration did not differ significantly between remimazolam and any comparator (*vs*. propofol: MD 1.18 min, *P* = 0.97; *vs*. dexmedetomidine: MD −0.08 min, *P* = 0.70; *vs*. midazolam: MD −2.26 min, *P* = 0.13) (Figs. 4B, 4F and 4J), though propofol and midazolam comparisons exhibited high heterogeneity (I^2^ ≥ 95%). For patient satisfaction, remimazolam and propofol achieved equivalent scores (MD −0.09, 95% CI [−0.26 to 0.07]; *P* = 0.25; I^2^ = 5%; representative scores 9.4 *vs*. 9.6/10) (Fig. 4H), while results against dexmedetomidine were inconclusive (MD 0.47, *P* = 0.12; I^2^ = 99%) (Fig. 4D), and midazolam data were unavailable.

### Incidence of adverse events

Compared to dexmedetomidine, remimazolam significantly reduced hypoxemia risk (OR 0.41, 95% CI [0.18–0.96]; *P* = 0.04) but increased tachycardia incidence (OR 3.01, 95% CI [1.37–6.60]; *P* = 0.006), with no differences in hypertension (OR 0.80, 95% CI [0.42–1.54]; *P* = 0.51) and inconclusive results for bradycardia/hypotension due to high heterogeneity (I^2^ > 75%). *vs*. propofol, remimazolam exhibited a marked reduction in hypotension (OR 0.35, 95% CI [0.23–0.55]; *P* < 0.00001) and a non-significant trend toward lower bradycardia risk (OR 0.33, 95% CI [0.09–1.18]; *P* = 0.08), while hypertension (OR 1.80, 95% CI [0.83–3.89]; *P* = 0.14) and tachycardia (OR 0.58, 95% CI [0.40–2.37]; *P* = 0.96) showed no significant differences. Against midazolam, no outcome differences reached significance: hypotension (OR 0.75, 95% CI [0.45–1.26]; *P* = 0.28), hypoxemia (OR 1.19, 95% CI [0.63–2.27]; *P* = 0.69), or tachycardia (OR 1.35, 95% CI [0.27–6.74]; *P* = 0.71), with uniformly low heterogeneity (I^2^ = 0%). Heterogeneity ranged from minimal (I^2^ = 0%) to substantial (I^2^ = 93%), and precision limitations were noted in wide confidence intervals.

## Discussion

This meta-analysis provides several novel contributions to the existing body of evidence. While previous reviews have established the general efficacy and safety of remimazolam, our study offers a more granular and contemporary synthesis specifically focused on bronchoscopy, directly comparing remimazolam against all three major sedative alternatives (propofol, dexmedetomidine, and midazolam) within a single analytical framework. This approach allows for a clear, hierarchical ranking of sedative performance for this specific procedure. Furthermore, we provide robust quantitative evidence on key outcomes like procedural success rates where previous data were limited or conflicting, particularly against dexmedetomidine and midazolam. Our analysis also delivers the most comprehensive safety profile to date, clearly delineating remimazolam’s superior hemodynamic stability compared to propofol—a finding of paramount clinical importance—and identifying its distinct adverse event pattern compared to dexmedetomidine (reduced hypoxemia but increased tachycardia).

This comprehensive meta-analysis of 13 randomized controlled trials, encompassing 2,002 patients, provides robust evidence regarding the comparative efficacy and safety of the novel ultra-short-acting benzodiazepine, remimazolam, for sedation during bronchoscopy. The findings illuminate remimazolam’s distinct profile compared to established sedatives—dexmedetomidine, propofol, and midazolam—across critical procedural and patient-centered outcomes, while also highlighting areas requiring careful clinical consideration and further research.

Beyond the direct drug comparisons, several clinical factors and methodological considerations could have influenced the observed outcomes (Granholm et al., 2022). The depth of sedation, often targeting a specific Modified Observer’s Assessment of Alertness/Sedation (MOAA/S) score across studies, is a critical variable. While most protocols aimed for moderate to deep sedation, subtle inter-study differences in the target depth or the timing of drug titration relative to procedural stimuli could affect both success rates and the incidence of adverse events like hypotension or hypoxemia (Fuller et al., 2022). Furthermore, heterogeneity in patient demographics and clinical status, partially addressed by our random-effects model, must be considered. Variables such as advanced age, pre-existing cardiopulmonary comorbidities, ASA physical status, and baseline opioid or benzodiazepine use could modulate individual responses to sedatives (Ceric et al., 2025), potentially explaining some of the heterogeneity in outcomes like hemodynamic events or recovery times (Ceric et al., 2024). For instance, the superior hemodynamic stability of remimazolam over propofol might be particularly pronounced in elderly or hemodynamically vulnerable patients, a subgroup warranting future investigation.

The most compelling finding is remimazolam’s significant superiority over both dexmedetomidine and midazolam in achieving procedural success—defined as the completion of the intended bronchoscopy without rescue sedation. The magnitude of this advantage is particularly striking against midazolam, with remimazolam achieving a 77.1% success rate compared to a remarkably low 3.6% for midazolam. This vast absolute difference underscores a fundamental limitation of midazolam in this context, likely attributable to its slower onset, variable pharmacokinetics, and potential for inadequate sedation depth or prolonged recovery leading to procedural interruption or abandonment (Prommer, 2020; Zaporowska-Stachowiak et al., 2019). While the comparison with dexmedetomidine also favored remimazolam, the moderate heterogeneity suggests that factors like dosing strategies or specific patient subgroups may modulate this effect. Crucially, remimazolam demonstrated equivalent procedural success rates to propofol. This parity is significant, establishing remimazolam as equally effective as the current gold standard propofol for ensuring procedure completion. The high success rates with both agents reinforce their reliability for bronchoscopists.

Remimazolam demonstrates a favorable pharmacokinetic profile translating into practical efficiency. Its significantly faster onset compared to dexmedetomidine aligns with expectations based on its mechanism of action (Tsukimoto et al., 2024). While statistically significant differences weren’t found *vs*. propofol or midazolam for onset time, the point estimates and direction of effect suggest remimazolam may be at least as fast as propofol and potentially faster than midazolam. The heterogeneity in these comparisons warrants caution in over-interpretation, but the lack of a clinically meaningful delay compared to propofol is reassuring. More impactful is remimazolam’s advantage in recovery time. Patients sedated with remimazolam woke up significantly faster than those receiving dexmedetomidine or midazolam. This rapid offset, stemming from its esterase-based metabolism independent of organ function, is a major clinical asset. It facilitates quicker patient assessment post-procedure, potentially increases throughput in busy endoscopy units, and allows for faster discharge in outpatient settings, enhancing operational efficiency and patient convenience (Io et al., 2021; Xiao et al., 2022). The comparison with propofol was inconclusive due to extreme heterogeneity, reflecting variations in propofol administration (bolus *vs*. TCI, dosing) and recovery definitions across studies. Future head-to-head trials with standardized protocols are needed to definitively compare recovery profiles.

Patient satisfaction is paramount in procedural sedation. This analysis found no significant difference in satisfaction scores between remimazolam and propofol. This high level of equivalent satisfaction, coupled with comparable procedural success, positions remimazolam favorably against the benchmark propofol from the patient’s perspective (An et al., 2025; Li et al., 2023). Data comparing satisfaction with dexmedetomidine was inconclusive, and no studies reported satisfaction scores *vs*. midazolam, representing a gap in the current evidence base that future studies should address.

Remimazolam’s most significant safety advantage lies in its significantly reduced risk of hypotension compared to propofol (Choi, Jang & Park, 2023). This represents a 65% relative risk reduction—a crucial finding given the high prevalence of comorbidities like cardiovascular disease in the bronchoscopy population. Propofol’s dose-dependent cardiovascular depression is a well-known limitation, often necessitating cautious titration and sometimes fluid resuscitation or vasopressors. Remimazolam’s hemodynamic stability offers a safer alternative, particularly for frail, elderly, or hemodynamically compromised patients. A non-significant trend towards lower bradycardia risk with remimazolam was also observed. Remimazolam significantly reduced the risk of hypoxemia compared to dexmedetomidine. This finding, though based on a limited number of studies, suggests remimazolam may offer better respiratory stability than dexmedetomidine during bronchoscopy, a procedure inherently challenging to oxygenation and ventilation.

The analysis indicated a significantly higher incidence of tachycardia with remimazolam compared to dexmedetomidine. While the clinical significance of transient tachycardia during a short procedure requires further evaluation, it warrants attention, especially in patients with underlying coronary artery disease or heart failure. The mechanism is unclear but could relate to differences in sympathetic modulation or baroreflex responses between the drugs.

No significant differences were found between remimazolam and midazolam regarding hypotension, hypoxemia, or tachycardia. The uniformly low heterogeneity in these comparisons strengthens this finding of comparable safety between these two benzodiazepines for these specific events, with remimazolam’s advantage lying primarily in efficacy and recovery speed.

The observed heterogeneity in the propofol comparisons, which led to inconclusive results for some outcomes, merits further discussion. Firstly, the relatively limited number of direct comparisons between remimazolam and propofol can be attributed to remimazolam’s more recent introduction to the market. While propofol has been a gold standard for decades, remimazolam is a novel agent, and thus the body of RCT evidence is still accumulating. Secondly, the inconsistency in dosing, modes of administration (*e.g*., bolus *vs*. target-controlled infusion for propofol), and definitions of recovery time (*e.g*., time to eye-opening, obeying commands, or Aldrete score ≥9) across studies reflects real-world variations in clinical protocols and research methodologies between different institutions and countries. These factors are inherent challenges in meta-analyses of clinical practice and are key contributors to the statistical heterogeneity we observed.

This study has several limitations that should be acknowledged. First, significant heterogeneity was observed in some comparisons, particularly among the propofol studies regarding recovery times and some adverse events. This variability likely stems from differences in propofol administration protocols (*e.g*., bolus *vs*. target-controlled infusion, dosing regimens), patient characteristics, and definitions of outcomes across the included trials, which could influence the pooled estimates. Second, the evidence base for certain comparisons remains restricted. The number of available RCTs was limited for comparisons against dexmedetomidine (four RCTs) and midazolam (three RCTs), constraining the statistical power for subgroup analyses and the robust assessment of less common adverse events. Furthermore, three identified propofol RCTs had to be excluded from the primary outcome meta-analysis due to unreported data, potentially affecting the comprehensiveness of that specific finding. Finally, as a study-level meta-analysis of aggregate data, our work is inherently secondary in nature. We were unable to access individual patient data, which precluded more nuanced analyses based on patient-level covariates (*e.g*., specific comorbidities, precise age strata) that might modify the treatment effects. Notwithstanding these limitations, we employed random-effects models to account for heterogeneity and have interpreted the findings, especially those with substantial heterogeneity or based on few studies, with appropriate caution.

This meta-analysis establishes remimazolam as a highly effective and safe sedative for bronchoscopy. Its rapid onset, predictable pharmacokinetics, availability of reversal, and significantly faster recovery compared to dexmedetomidine and midazolam, coupled with procedural success rates matching propofol and superior hemodynamic stability, position it as a valuable addition to the bronchoscopist’s armamentarium. It offers a compelling alternative to midazolam, overcoming its key limitations of inadequate sedation and slow recovery. Compared to propofol, it provides equivalent efficacy and patient satisfaction with significantly improved cardiovascular tolerability, making it particularly suitable for vulnerable populations.

The clinical relevance of these findings is substantial. First, for clinicians seeking an alternative to propofol, particularly in settings where anesthesia support is limited or for patients prone to hypotension (*e.g*., the elderly, those with cardiovascular comorbidities), remimazolam presents a compelling option with equivalent efficacy and superior hemodynamic stability. Second, our data strongly support replacing midazolam with remimazolam for bronchoscopy, given the dramatic improvement in procedural success and recovery speed without compromising safety. The comparison with dexmedetomidine is more nuanced; the choice may depend on whether faster onset/recovery (favoring remimazolam) or avoidance of tachycardia (favoring dexmedetomidine) is prioritized for a given patient. This synthesis thus moves beyond establishing non-inferiority and provides practical, evidence-based guidance for individualized sedative selection in bronchoscopy suites.

## Conclusions

Based on our findings, remimazolam is strongly recommended as a first-line sedative for bronchoscopy, particularly in hemodynamically vulnerable patients (*e.g*., elderly or those with cardiovascular instability) where it provides propofol-equivalent efficacy with significantly reduced hypotension risk. Its faster recovery profile also makes it ideal for outpatient settings, improving throughput compared to dexmedetomidine and midazolam. When choosing between remimazolam and dexmedetomidine, prioritize the former for faster onset/recovery and the latter when avoiding tachycardia is critical. Remimazolam is clearly superior to midazolam for routine use, addressing the latter’s limitations of inadequate sedation and prolonged recovery. Standardized monitoring for tachycardia is advised when using remimazolam, especially in patients with cardiac disease.

In conclusion, remimazolam emerges from this synthesis as a versatile and advantageous sedative for bronchoscopy, combining high efficacy, rapid recovery, and a favorable safety profile, particularly characterized by hemodynamic stability relative to propofol. The evidence supports its consideration as a first-line sedative agent for a broad range of patients undergoing this procedure. Its distinct pharmacokinetic and safety profile facilitates a more personalized approach to sedation, allowing clinicians to tailor their choice based on patient comorbidities and procedural priorities. It represents a significant step towards the ideal sedative profile for this common and essential respiratory procedure.

## Supplemental Information

10.7717/peerj.20552/supp-1 Supplemental Information 1 PRISMA checklist.

10.7717/peerj.20552/supp-2 Supplemental Information 2 Characteristics of included randomized controlled trials comparing remimazolam with other sedatives for bronchoscopy sedation.

10.7717/peerj.20552/supp-3 Supplemental Information 3 Raw data.

## Additional Information and Declarations

### Data Availability

The following information was supplied regarding data availability:

Raw data is available in the Supplemental Files.
